# Supplementary material for: Differential Stress Responses to Rice Blast Fungal Infection Associated with the Vegetative Growth Phase in Rice
Source: Plants (Basel). 2025 Jan 16;14(2):241. doi: 10.3390/plants14020241 (PMC11769066; doi:10.3390/plants14020241)
Supplement: Supplementary file 1 [file plants-14-00241-s001.zip › Supplementary Figures.pdf]

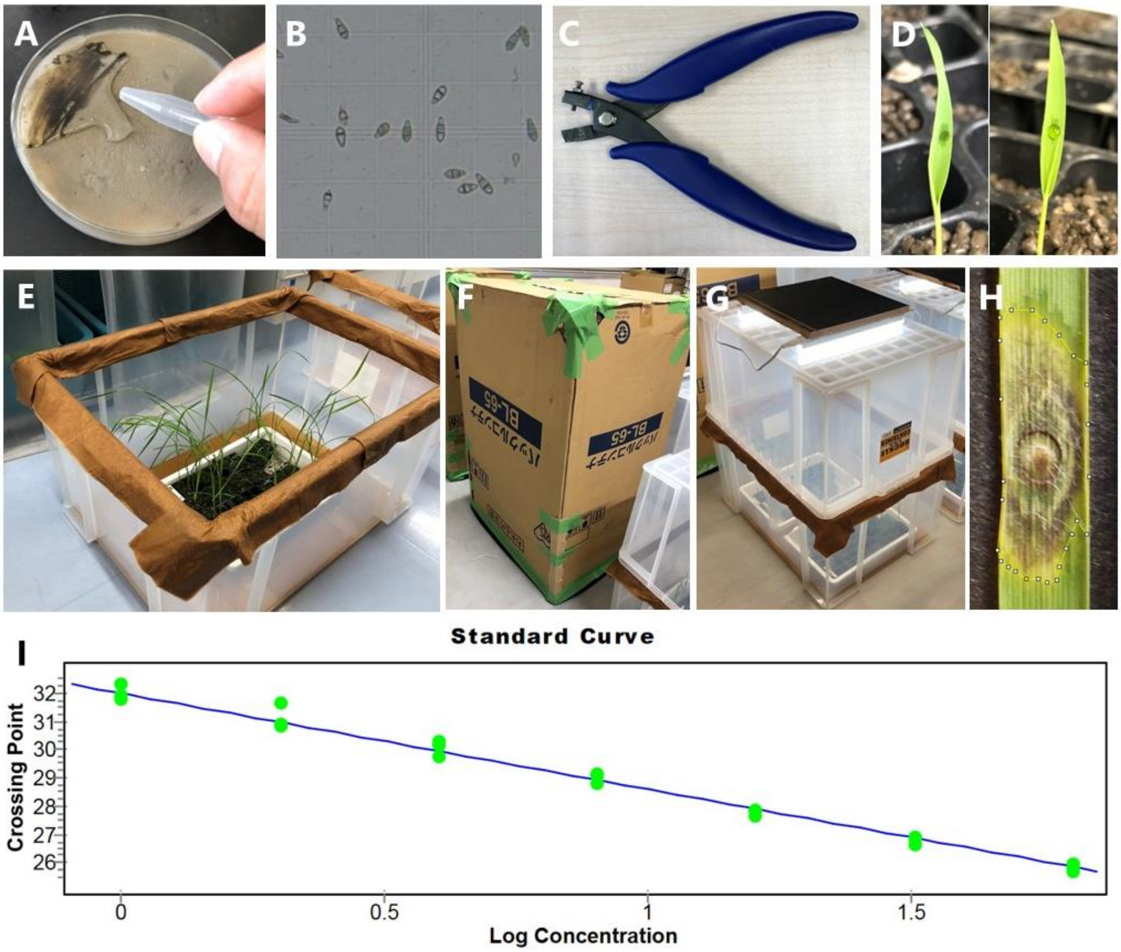

Supplementary Figure S1. Rice blast conidia inoculation method and disease severity evaluation method.

(A) *M. oryzae* conidia formed on oatmeal medium were collected by adding water and scratching them at the bottom of the tube. (B) *M. oryzae* conidia on a hemocytometer. (C) The tool used in this experiment for wounding leaf blades. Wounds were made by pressing leaves with the flatten bottom of the screw attached to the tool. (D) A wound made on the second leaf (left) and 3 µl of conidial suspension applied on the wound (right). (E) A plastic box to create 100% humidity condition. Paper towels were spread in the box and bottom of the box was filled with water. Then, the box was covered with another plastic box. (F) A cardboard box for creating complete dark condition. Humid boxes containing inoculated plants were covered with cardboard boxes for 1 day. (G) Inoculated plants under light condition. Inoculated plants were kept in the dark for 1 day and then maintained under a 14-h light/10-h dark cycle until disease severity assessment. (H) Lesion area measurement with imageJ. Each lesion area was surrounded with a polygon by hand to measure area. (I) Standard curve used for fungal mass measurement. Relative fungal mass in each sample was calculated based on this standard curve and machine-calculated Cp values.

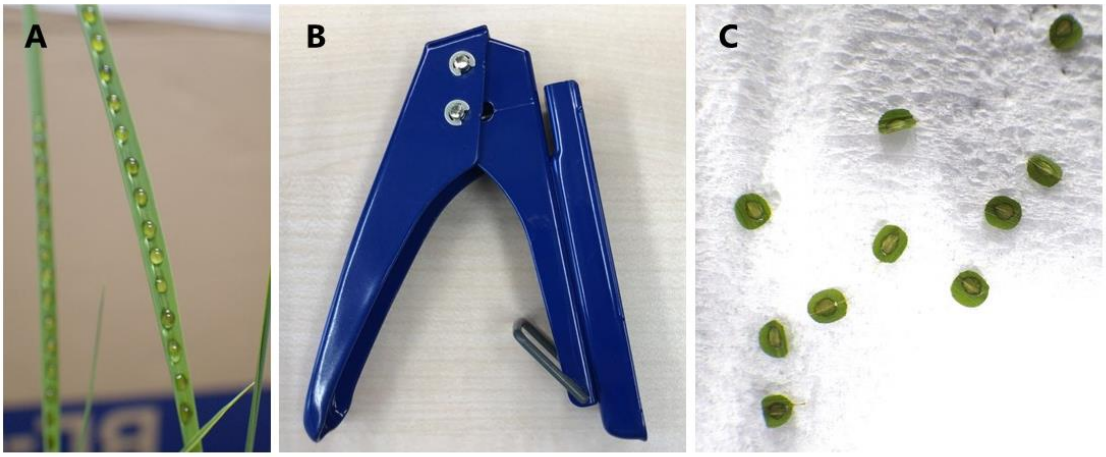

Supplementary Figure S2. Inoculation and sampling method for RNA-seq.
(A) Wounds with 3 µl of conidial suspension. 3 to 16 spots were prepared on each leaf to extract enough amount of RNA from one leaf. (B) A hole puncher used for sampling. (C) Collected leaf discs. Leaf discs around each wound were sampled and immediately frozen in liquid nitrogen.

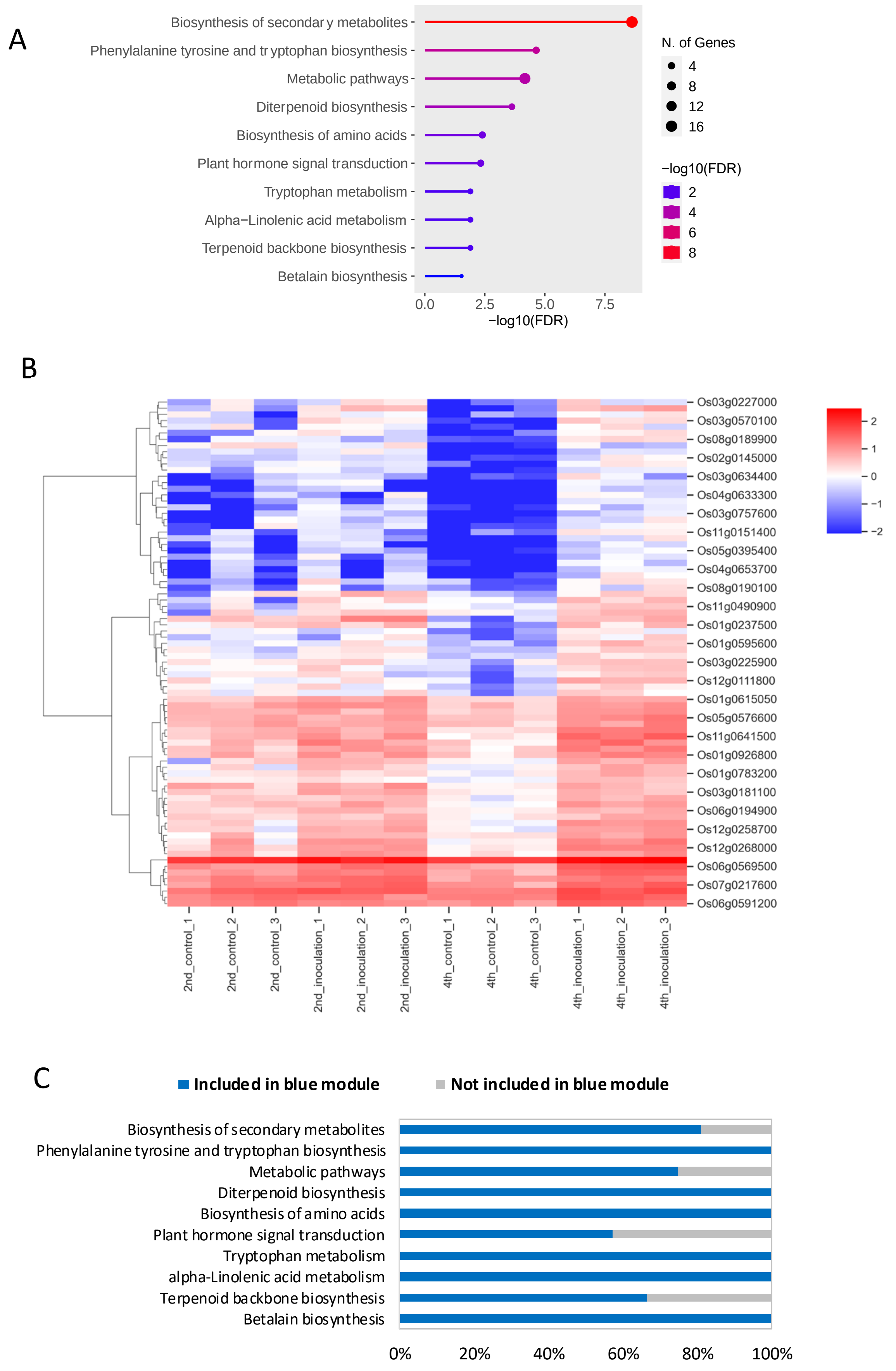

Supplementary Figure S3. Clustering and GO enrichment analysis of DEGs in the blue module.

(A) GO enrichment analysis of 82 genes in the blue module. (B) Clustering analysis of 82 genes in the blue module. (C) Percentage of genes in the blue module among DEGs up-regulated in the fourth leaves for each GO term.

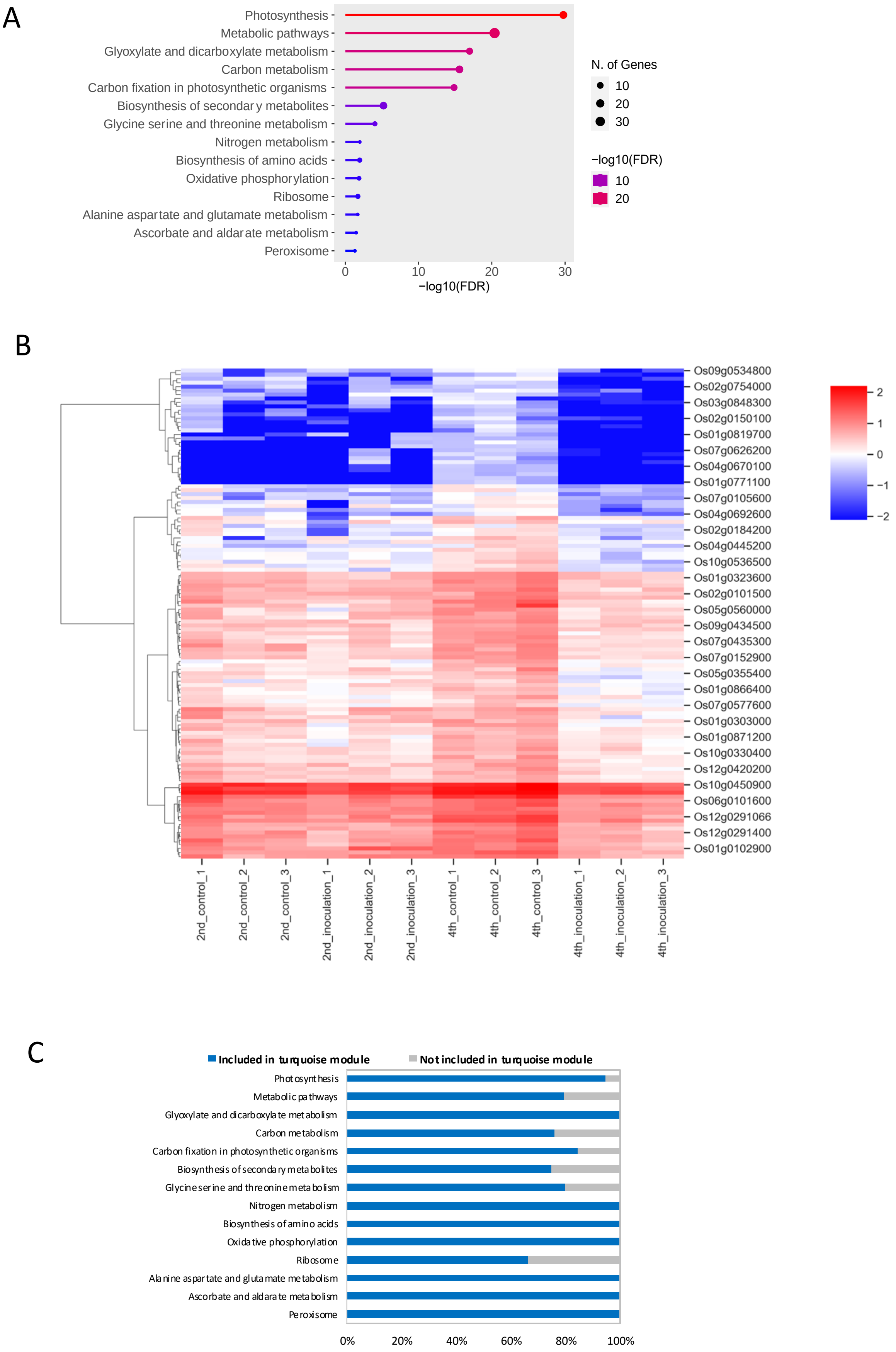

Supplementary Figure S4. Clustering and GO enrichment analysis of DEGs in the turquoise module.

(A) GO enrichment analysis of 123 genes in the turquoise module. (B) Clustering analysis of 123 genes in the turquoise module. (C) Percentage of genes in the turquoise module among DEGs down-regulated in the fourth leaves for each GO term.
